# Glances at the Hospitals

**Published:** 1901-04-27

**Authors:** 


### GLANCES AT THE HOSPITALS.

### THE PETERBOROUGH INFIRMARY AND DISPENSARY.

The total income for the year was £2,096 and the total expenditure was £2,131. The number of in-patients was 509, and the total number of patients treated and discharged during the year was 4,353. Of these 3,522 were cured, 268 relieved, 29 incurables, 64 died, surgical appliances given to 290, and 174 were discharged for various reasons. The tables are carefully prepared, but it would be better if in-patients' and out-patients' statistics were given on separate pages. There does not seem to be any good reason for mixing these two classes of patients. From the Physicians' Report, however, we learn that there were 175 medical in-patients, and of these 101 were cured, 41 relieved, and 13 died, the mortality being 7·6 per cent. The surgical in-patients numbered 334, and the mortality was 2·3 per cent. A table showing the anæsthetic used during the operations is given, and we note that ether was used 114 times, chloroform 99 times, and nitrous oxide 24 times. Locally the ether spray was employed 45 times, and cocaine 59 times. The average cost per bed occupied, the average cost per week, and the average residence in the hospital should be stated in future reports.

### THE WOLVERHAMPTON GENERAL HOSPITAL.

The total ordinary income of this hospital for the year 1899 was £9,401, and the total ordinary expenditure was £9,249. The income and expenditure are both a little higher than they were for 1898; but it is as pleasing, as it is unusual, to be able to chronicle the administration of a hospital in which the balance is on the right side. The extraordinary expenditure amounted only to £116; and it is now proposed to improve the operating theatre and to add electric light. There were 120 patients in the hospital on January 1st, 1899. During the year 2,092 new cases were admitted, making a total of 2,212. Of these 1,486 were cured, 329 relieved, 79 discharged for other reasons, 200 died, and 118 remained under treatment. The number of out-patients reached a total of 16,363. There were 512 operations performed in the theatre of the hospital; and of these 422 were successful and 23 died, and the remaining 67 being relieved or unrelieved. The medical and surgical tables are well arranged, and convey all necessary information. A short table showing the nature of the anæsthetics used would be a desirable addition to next year's report.

### THE GENERAL INFIRMARY, GLOUCESTER.

The General Committee say that the financial condition of the infirmary causes them much anxiety because there are increasing claims on the hospital without a corresponding increase of income, and when we notice that at the close of 1899 there was a deficit of £960 we can easily understand the anxiety, more especially as both the preceding years showed deficits, although not to such a serious extent. The total income amounted to £8,487, and of this the workpeople contributed £468. On January 1st, 1899, there were 113 in-patients, and 1,328 were admitted during the year, making a total of 1,441. Of these 880 were cured, 349 relieved, 45 not relieved, 75 died, and 92 remained under treatment. The total number of out-patients was 8,187; and 7,908 of these were discharged cured or relieved, 279 remaining under treatment. The medical and surgical tables are very carefully compiled, and separate columns show the numbers of each disease cured, relieved, not relieved, and died. Anæsthetics were administered 494 times. In 372 of these ether was the agent, in 106 chloroform, and in 16 nitrous oxide. The out-patients had ether 33 times, chloroform 36, and nitrous oxide 54.

